# Bispectral Index Alterations and Associations With Autonomic Changes During Hypnosis in Trauma Center Researchers: Formative Evaluation Study

**DOI:** 10.2196/24044

**Published:** 2021-05-26

**Authors:** C Michael Dunham, Amanda J Burger, Barbara M Hileman, Elisha A Chance, Amy E Hutchinson

**Affiliations:** 1 St Elizabeth Youngstown Hospital Youngstown, OH United States

**Keywords:** bispectral index, hypnosis, heart rate variability, electromyography, skin conductance, skin temperature, respiratory rate, expired carbon dioxide, neurofeedback

## Abstract

**Background:**

Previous work performed by our group demonstrated that intermittent reductions in bispectral index (BIS) values were found during neurofeedback following mindfulness instructions. Hypnosis was induced to enhance reductions in BIS values.

**Objective:**

This study aims to assess physiologic relaxation and explore its associations with BIS values using autonomic monitoring.

**Methods:**

Each session consisted of reading a 4-minute baseline neutral script and playing an 18-minute hypnosis tape to 3 researchers involved in the BIS neurofeedback study. In addition to BIS monitoring, autonomic monitoring was performed, and this included measures of electromyography (EMG), skin temperature, skin conductance, respiratory rate, expired carbon dioxide, and heart rate variability. The resulting data were analyzed using two-tailed *t* tests, correlation analyses, and multivariate linear regression analyses.

**Results:**

We found that hypnosis was associated with reductions in BIS (*P*<.001), EMG (*P*<.001), respiratory rate (*P*<.001), skin conductance (*P*=.006), and very low frequency power (*P*=.04); it was also associated with increases in expired carbon dioxide (*P*<.001), skin temperature (*P*=.04), high frequency power (*P*<.001), and successive heart interbeat interval difference (*P*=.04) values. Decreased BIS values were associated with reduced EMG measures (R=0.76; *P*<.001), respiratory rate (R=0.35; *P*=.004), skin conductance (R=0.57; *P*<.001), and low frequency power (R=0.32; *P*=.01) and with increased high frequency power (R=−0.53; *P*<.001), successive heart interbeat interval difference (R=−0.32; *P*=.009), and heart interbeat interval SD (R=−0.26; *P*=.04) values.

**Conclusions:**

Hypnosis appeared to induce mental and physical relaxation, enhance parasympathetic neural activation, and attenuate sympathetic nervous system activity, changes that were associated with BIS values. Findings from this preliminary formative evaluation suggest that the current hypnosis model may be useful for assessing autonomic physiological associations with changes in BIS values, thus motivating us to proceed with a larger investigation in trauma center nurses and physicians.

## Introduction

### Effects of Hypnosis on Brainwave Physiology

Multiple investigations have shown that a hypnotic state can influence alterations in brainwave activity. Specifically, studies have provided evidence that hypnosis reduces beta brainwave power [1-3]. In addition, multiple studies have shown that hypnosis is associated with increased theta brainwave power [4-6] and enhanced alpha brainwave power [1,6]. These brainwave alterations are indicative of a state of mental relaxation. Of particular relevance are investigations demonstrating that bispectral index (BIS) values substantially decrease during nonpharmacologic hypnosis [7,8]. Other investigators have provided evidence of reductions in BIS values for participants who listened to relaxing music [9], watched relaxing videos [10], or underwent relaxing-guided imagery [11].

### Effects of Hypnosis on Heart Rate Variability

When measuring the electrocardiographic R-to-R interval in milliseconds, there is a normal variation from one beat to the next in healthy participants [12,13]. Standard measures of heart rate variability (HRV) include the SD of cardiac interbeat (normal-to-normal; SDNN) time intervals and root mean square of successive cardiac interbeat time intervals (RMSSD). Common HRV measurements also include spectral density measurements of high frequency (HF) power, low frequency (LF) power, very low frequency (VLF) power, LF/HF power ratio, and total power [12,13]. LF power has been shown to be influenced by sympathetic and parasympathetic nervous system activities, whereas HF power better reflects parasympathetic activation [12-15]. Multiple investigators have explored the influence of hypnosis on HRV. With hypnosis, LF power has been shown to decrease [16-18], whereas HF power has been demonstrated to increase [17,19,20]. Accordingly, LF/HF power has been noted to decrease [16,21]. During hypnosis, RMSSD, SDNN, and the interbeat interval mean and SD have been shown to increase [19,20].

### Effects of Stress-Relaxation on HRV

Several investigators have demonstrated the effects of stress and relaxation on HRV. Improvements in HRV measurements have been shown in autonomic balance during relaxation therapies and interventions [10,22,23], mindfulness interventions [24], and laparoscopic surgeries with robotic assistance [25]. HRV has also been shown to decrease with psychological stress [15,26], perceived high work stress in nurses [27], and stress in nursing students [28].

### Effects of Hypnosis on Other Autonomic Physiology

Several studies have documented the effects of hypnosis on skin conductance, expired carbon dioxide, respiratory rate, electromyography (EMG), and skin temperature values. Two relatively recent studies have demonstrated that hypnosis is associated with reductions in skin conductance values [8,29]. We found that only a single study investigated expired carbon dioxide measurements during hypnosis [30]. The data in the manuscript showed that expired carbon dioxide levels increased and respiratory rates decreased; however, details of the statistical analysis were not provided. Significant reductions in the respiratory rate have been shown to occur with hypnosis [31,32]. Hypnosis has been demonstrated to reduce EMG tension in the masseter, temporalis, and frontalis muscles [33-35]. Virtually all studies in the literature that assessed the effects of hypnosis on skin temperature involved volitional efforts to alter the temperature or to mitigate temperature responses to heat or cold stressors. However, we found an investigation demonstrating that hypnosis, without any intent to change temperature, was associated with a significant increase in finger temperature [36].

### Effects of Stress-Relaxation on Other Autonomic Physiology

Peripheral skin temperature is mediated according to autonomic nervous system regulation; that is, temperature reductions occur with increases in sympathetic neural activity [37]. Reductions in peripheral skin temperature have been associated with psychological stress [38] and mental stress [39,40]. On the other hand, biofeedback relaxation has been found to be associated with increases in peripheral skin temperature [41]. Electrical skin conductance values increase with sympathetic neutrally mediated palmar sweating [37]; multiple investigations have shown that acute mental stress is associated with increases in electrical skin conductance values [42-46]. Evidence in the literature shows that decreased BIS values are associated with decreased skin conductance [47].

Respiratory rate increases with anxiety, whereas mindfulness and relaxation tend to decrease ventilation effort [37]. Investigations have demonstrated that respiratory rates increase with acute mental stress [48-52] and acute fear [53]. In contrast, relaxing music [9], progressive muscle relaxation or galvanic skin resistance biofeedback [54], and yoga training [55] have been associated with significant reductions in respiratory rates. Generally, the expired carbon dioxide concentration has an inverse relationship with the respiratory rate [37]. Expired carbon dioxide has been found to significantly decrease in participants with emotional disorders [56], generalized anxiety disorder [57], acute fear [53], and acute mental stress [50,58]. In contrast, increased expired carbon dioxide has been found to occur with yoga training [55] and progressive muscle relaxation [48].

Surface EMG measures muscle tension beneath the skin at designated anatomical sites [37]. Increased surface EMG activity has been associated with acute mental stress [59], viewing distressing videos [60], and perceptions of negative stress states [61]. Conversely, reduced surface EMG activity has been linked to relaxation interventions [62], relaxation training [63], and meditation [64].

### BIS and Monitoring of Coparameters

The National Library of Medicine contains 2607 citations regarding the use of BIS monitoring in humans. Some of these manuscripts describe simultaneous changes in BIS values, HRV, skin conductance, respiratory rate, expired carbon dioxide, and EMG values; however, a smaller number of studies presented correlation analyses between BIS values and these other parameters. The limited relevance of these investigations is that multiple confounding conditions typically exist during each investigation. For example, study participants usually undergo substantial physical stimulation, such as invasive surgery, airway manipulation, or mechanical ventilation. Often, the research includes participants with brain pathologies, such as traumatic brain injury, cerebrovascular diseases, seizure disorders, or coma. Furthermore, most study participants in these investigations are administered intravenous or inhalation anesthesia, intravenous sedatives, or neuromuscular blocking agents.

Several studies have provided simultaneous measurements of HRV and BIS values; however, these investigations are substantially confounded by multiple factors [65-68]. A study of participants who watched relaxing videos had concomitant decreases in BIS values and increments in HF power measurements, but correlation data between BIS and HF power were not shown [10]. A significant correlation has been found between BIS and EMG values, but this observation was relevant to intraoperative patients [69]. In addition, EMG activity has been demonstrated to contribute considerably to high BIS values in postoperative patients [70].

BIS and respiratory rate values have been shown to simultaneously decrease during music therapy in intensive care unit patients [9]. A study compared simultaneous BIS values, respiratory rate, and expired carbon dioxide measurements in healthy volunteers, but these findings were confounded by the application of a tight-fitting facemask and continuous infusions of intravenous sedatives [71]. Another study monitored serial BIS values, respiratory rate, and expired carbon dioxide measurements; however, these findings were confounded by the need for cataract surgery in moderately sedated participants with varying levels of health status [72]. In a single study, BIS and skin conductance were shown to significantly decrease during hypnosis; however, the authors provided no correlation analysis between the 2 values [8]. In addition, another study demonstrated that BIS and skin conductance values were positively correlated in patients undergoing surgical anesthesia [47].

### BIS Neurofeedback Study

Our group demonstrated that trauma center nurses and physician participants could learn to self-regulate brainwave activity using an electroencephalography (EEG)-based BIS monitoring system during neurofeedback immediately after receiving mindfulness instructions [73]. Importantly, most participants also showed improvements in well-being scores after learning brainwave self-regulation. These findings serve as a validation indicator that using the BIS monitor to perform brainwave self-regulation during neurofeedback can be useful. During the 228 neurofeedback learning sessions, participants demonstrated the ability to lower their BIS values following mindfulness instruction (brainwave self-regulation). However, the reductions were often relatively brief; they returned near the baseline value and subsequently decreased again.

First, we sought other evidence to validate the notion that the BIS monitor is a potentially useful tool for performing neurofeedback. In particular, we wanted to use a methodological approach that would likely produce sustained reductions in BIS values. Second, we wanted to use ancillary physiologic monitoring device data that we could simultaneously compare with BIS values. Our goals were to determine whether hypnosis could produce sustained reductions in BIS values and to organize a process evaluation that would potentially demonstrate that reductions in EEG-based BIS values are associated with a physiological state of relaxation.

### Aims

The aims of this feasibility and formative assessment are two-fold. First, we aim to use hypnosis and to describe significant changes that occurred in BIS, HRV, skin conductance, expired carbon dioxide, respiratory rate, EMG, and skin temperature values. Second, we intend to identify any significant relationships between BIS measurements and autonomic physiologic variable values. If the formative assessment appears to be meritorious, we plan to apply a similar approach and process to a larger group of physicians and nurses employed at the same trauma center.

## Methods

### Assessment Design and Population

This formative assessment was performed in accordance with the recommendations of the Declaration of Helsinki. The protocol was approved by St Elizabeth Youngstown Hospital, Mercy Health Youngstown, LLC’s institutional review board (institutional review board organization #0001624). On November 21, 2019, the review board granted expedited approval, and because the evaluation was deemed to have minimal risk, they waived consent (institutional review board approval number: 19-024). Three of the trauma center research investigators (healthy volunteers) who conducted the BIS neurofeedback study participated as participants in this evaluation [73]. There was no intention to compare results between the participants. We intended to compare prehypnotic data with active hypnotic data to determine whether hypnosis could produce sustained reductions in BIS values and whether BIS values are associated with other physiologic measurements of relaxation, forming an approach and process for applying this protocol to a larger group of trauma center physicians and nurses.

### Data Variables

Data variables assessed during this evaluation included BIS, EMG (decibels), expired carbon dioxide (mm Hg), respiratory rate (breaths per minute), skin temperature (degrees Fahrenheit), skin conductance (microsiemens), and HRV values. The specific HRV variables were (1) SDNN, (2) RMSSD, (3) LF power, (4) HF power, (5) VLF power, (6) relative LF power (LF power/total power), (7) relative HF power (HF power/total power), and (8) relative VLF power (VLF power/total power). LF power was the absolute power at a band frequency of 0.04-0.15 Hz and measured as ms^2^/Hz. HF power was the absolute power at a band frequency of 0.15-0.4 Hz and measured as ms^2^/Hz. VLF power was the absolute power at a band frequency of 0.0033-0.04 Hz and measured as ms^2^/Hz. Relative LF, HF, and VLF power were quantified as percentages of the total power at the relevant band frequencies. The potential BIS value range of the system is 0-100; however, awake and alert values typically approach 100 [73].

### Signal Sensor Applications

The Bispectral Index Vista Monitoring System (Aspect Medical Systems, Inc) was used to capture BIS and EMG physiological signals. According to the manufacturer’s instructions, the BIS sensor was applied to the participant’s forehead and temporal fossa. The BIS sensor contained 4 electrodes that corresponded to the international 10-20 EEG system for electrode placement: FPz, FP1, AF7, and FT9. Frontalis muscle EMG activity was measured using the AF7 BIS sensor electrode. Monitoring of expired carbon dioxide respiratory rate was performed using the RespSense capnography monitor (model LS1R-9R, Nonin Medical, Inc). Each participant had a biprong nasal cannula inserted with tubing draped over the ears and was instructed to keep their lips closed.

The ProComp Infiniti encoder hardware system (Thought Technology Ltd) was used to capture skin temperature, skin conductance, and electrocardiographic signals (HRV variables). The skin temperature sensor was secured to the volar surface of the terminal phalanx of the middle finger using a Velcro strap. The 2 skin conductance sensors were secured to the volar surface of the middle phalanx of the ring and index fingers of the opposite hand with Velcro straps. Following the manufacturer’s instructions, 3 sensors were applied to the forearms to acquire electrocardiographic signals. The yellow sensor was attached to the volar side of the proximal right forearm. The black sensor was placed on the volar side of the proximal left forearm, and the blue sensor was attached 4 inches distally. Each participant’s arms were placed in a relaxed manner on chair armrests. Skin temperature, skin conductance, and electrocardiographic sensors were connected to the ProComp Infiniti encoder.

### Signal Processing

The Bispectral Index Vista Monitoring System (hardware and software) transformed BIS and EMG physiological signals into digital outputs. The RespSense capnography monitor, hardware and software, converted carbon dioxide measurements into a digital quantity. As each real-time peak expired carbon dioxide value represents a participant’s respiratory exhalation, the respiratory rate was computed by documenting the periodicity of carbon dioxide exhalations relative to time. The ProComp Infiniti encoder hardware system streamed physiological signal information to the BioGraph Infiniti (Thought Technology Ltd) Software, which transformed skin temperature, skin conductance, and electrocardiographic signals (HRV variables) into digital outputs.

### Signal Output Storage and Data Harvest

The BIS and EMG digital outputs were routinely captured on the hardware during each session. The cursor review monitor option provided a reading every 10 seconds. The investigators computed the mean BIS and EMG values for each session minute by averaging the 6 values for each minute. The values were entered into Microsoft Excel (Microsoft Corp.). The expired carbon dioxide and respiratory rate values were updated on the monitor hardware every second. The data were exported to Microsoft Excel, and a mean value for each session minute was computed by averaging the 60 expired carbon dioxide and respiratory rate values for each minute. The BioGraph Infiniti software computed and stored a 1-minute mean skin temperature and skin conductance result for each of the HRV variables. Accordingly, a mean 1-minute value was available for all physiological signals. These values were exported to Microsoft Excel, and then, all the data were imported into a statistical software program (SAS System for Windows, release 9.2, SAS Institute Inc).

### Signal Quality Assessments and Systems Coordination

Before starting the recording session, broadcasting the neutral script, and playing the hypnosis recording, the investigators examined the quality of the physiological signals. These assessments included an appraisal of the signal quality index and absence of artifacts as displayed on the BIS monitor. Capnography tracing and the stability of the expired carbon dioxide values were also examined. Finally, the researchers assessed the stability of skin temperature, skin conductance, and electrocardiographic tracings using the BioGraph Infiniti software system. The investigators coordinated the activation of the 3 monitoring systems with the initiation of a neutral script sound. As soon as the hypnosis recording ended, recordings within the 3 monitoring systems were immediately discontinued.

### Session Audio Scripts

During the 22-minute session, the participants were comfortably seated in an upright chair. The 22-minute session time was chosen to capture 4 minutes of prehypnotic or baseline values and 18 minutes of active hypnosis values. For the first 4 minutes of the session, a neutral script was read that contained neither stressful nor relaxing suggestions. The purpose of this script was to foster a cognitive state of alert, eyes-opened prehypnosis baseline to compare with the 18 minutes when the hypnotic script was played. Immediately following the conclusion of the neutral script, a hypnosis tape in MP3 format found in the public domain was played. While listening to the instructions of the hypnosis tape, the participants’ eyes were closed for most of the time. The script timeline milestones for each 22-minute session are presented in Table 1. A summary overview of the procedures performed during the experimental period is presented in Textbox 1.

**Table 1:** Audio script milestones.

Minute	Audio script	Comments
1	Neutral	Eyes open
2	Neutral	Eyes open
3	Neutral	Eyes open
4	Neutral	Eyes open
5	Start hypnosis	Eyes open
6	Physical relaxation	Eyes closed^a^
7	Physical relaxation	Eyes closed^a^
8	Deepening	Eyes closed^a^
9	Deepening	Eyes closed^a^
10	Mental relaxation	Eyes closed^a^
11	Mental relaxation	Eyes closed^a^
12	Mental relaxation	Eyes closed^a^
13	Deepening	Eyes closed^a^
14	Deepening	Eyes closed^a^
15	Deepening	Eyes closed^a^
16	Deepening	Eyes closed^a^
17	Deepening	Eyes closed^a^
18	Suggestions	Eyes closed^b^
19	Suggestions	Eyes closed^b^
20	Reorientation and awakening	Eyes closed
21	Reorientation and awakening	Eyes closed
22	Reorientation and awakening	Eyes open

^a^Infrequent, very brief prompts to open the eyes and then reclose them.

^b^“Every day in every way, I am getting better and better.”

**Procedural flow table.**

Sensor application (all applied per the manufacturer’s instructions)

- Sensors for bispectral index and electromyography applied to the participant’s forehead
- Carbon dioxide nasal cannula inserted
- Skin temperature sensor secured to the participant’s finger
- Skin conductance sensors secured to the participant’s fingers
- Electrocardiography sensors applied to the participant’s forearms

Sensor connections

- Sensors connected to the relevant hardware

Signal quality assurance

- Each monitor was assessed for signal quality

Session start

- Data recordings began at minute 1

Minutes 1-4, prehypnosis data

- Neutral script

Minute 5, hypnosis data

- Hypnosis tape started

Minutes 6-7

- Physical relaxation phase

Minutes 8-9

- Deepening of the physical relaxation phase

Minutes 10-12

- Mental relaxation phase

Minutes 13-17

- Deepening of mental and physical relaxation

Minutes 18-19

- Suggestive phase

Minutes 20-22

- Reorientation and awakening phase

Session end

- Hypnosis recording ended
- Data recordings ended
- Sensors removed from the participant
- Data entered into or exported to Microsoft Excel and analyzed with statistical software

### Statistical Analysis

Results were entered into an Excel 2010 worksheet (Microsoft Corp) and imported into the SAS System for Windows, release 9.2 (SAS Institute Inc). All mean values were accompanied by their SDs. For 2-group interval data comparisons, a two-tailed *t* test result and Cohen *d* were computed. Correlation analyses were assessed using the Pearson coefficient procedure. The level of significance was set at *P*<.05.

## Results

### Overview

Three healthy adult volunteers each completed a 22-minute experimental session. Of the 3 volunteers, there was 1 (33%) male participant and 2 (66%) female participants with ages ranging from 35 to 71 years. All participants were college graduates and single and received monthly compensation from a large hospital corporation. All participants verbalized a feeling of relaxation at the conclusion of their respective sessions.

### Autonomic Data Variances

The first 4-minute interparticipant variances were substantial for the following variables: expired carbon dioxide (mean 32.9, SD 5.1 mm Hg), respiratory rate (mean 16.9, SD 2.7 breaths per min), skin temperature (mean 91.4, SD 6.0 °F), skin conductance (mean 3.0, SD 2.1 microsiemens), LF power (mean 576, SD 488 ms^2^/Hz), HF power (mean 364, SD 301 ms^2^/Hz), VLF power (mean 433, SD 480 ms^2^/Hz), total power (mean 1372, SD 913 ms^2^/Hz), RMSSD (mean 54.2, SD 16.3 ms), and SDNN (mean 59.0, SD 17.9 ms). Owing to the variance sizes, a mean value was created from the first 4-minute raw values separately for each of these physiologic variables and each participant. Then, all the relevant 22-minute variable data results were divided by the physiologic variable mean value for the first 4 minutes for each participant; that is, all first 4-minute normalized variable values were approximately 1.0, whereas the normalized variable values for minutes 5-22 were relative fractions of 1.0, either ≥1.0 or <1.0 [19].

### Changes in Autonomic Variables During Hypnosis

**Figure 1:**
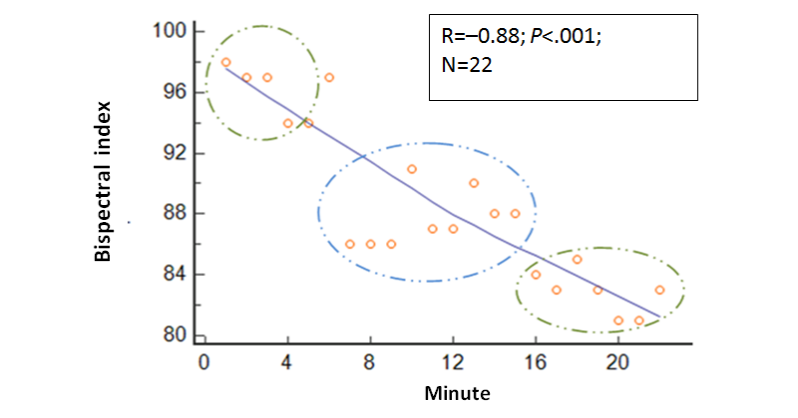
Relationship of the mean bispectral index with time. As the session progressed, the mean bispectral index values steadily decreased.

As the session progressed, the BIS values decreased and had a correlation between the 66 BIS values and the session duration in minutes (correlation analysis: raw BIS values=session minutes; R=−0.70; *P*<.001). A correlation existed between the 22 mean BIS values and the duration of the session in minutes (R=−0.88; *P*<.001; Figure 1). BIS values were lower for hypnosis minutes 7-15 (mean 87.7, SD 6.0) than for prehypnosis minutes 1-4 (mean 96.5, SD 1.7; *P*<.001; Cohen *d*=2). BIS values were also lower for hypnosis minutes 16-22 (mean 82.9, SD 4.1) than for hypnosis minutes 7-15 (mean 87.7, SD 6.0; *P*=.002; Cohen *d*=0.9).

**Figure 2:**
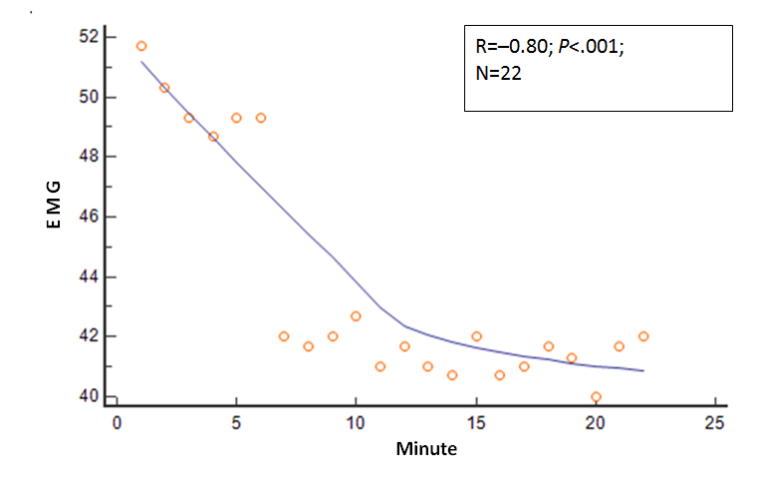
Relationship of the mean EMG with time. As the session progressed, the mean EMG values steadily decreased. EMG: electromyography.

**Figure 3:**
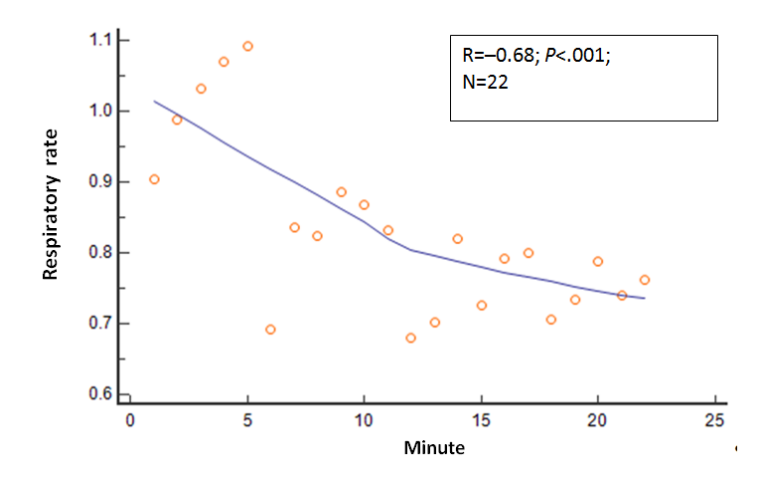
Relationship of the mean respiratory rate with time. As the session progressed, the mean respiratory rate values steadily decreased.

**Figure 4:**
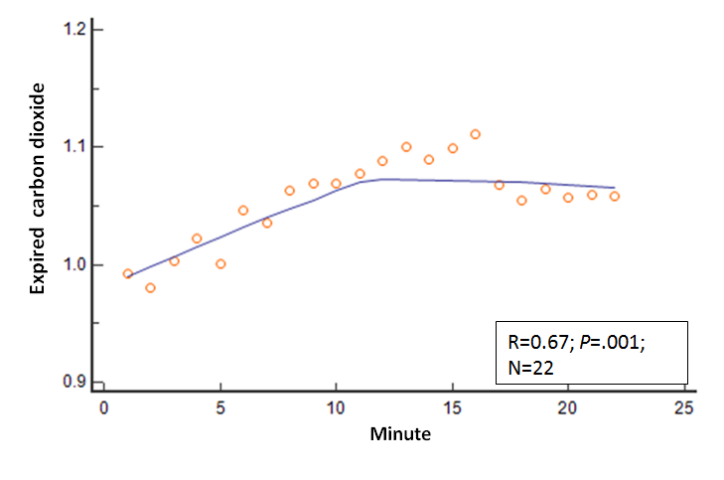
Relationship of the mean expired carbon dioxide with time. As the session progressed, the mean expired carbon dioxide values steadily increased.

**Figure 5:**
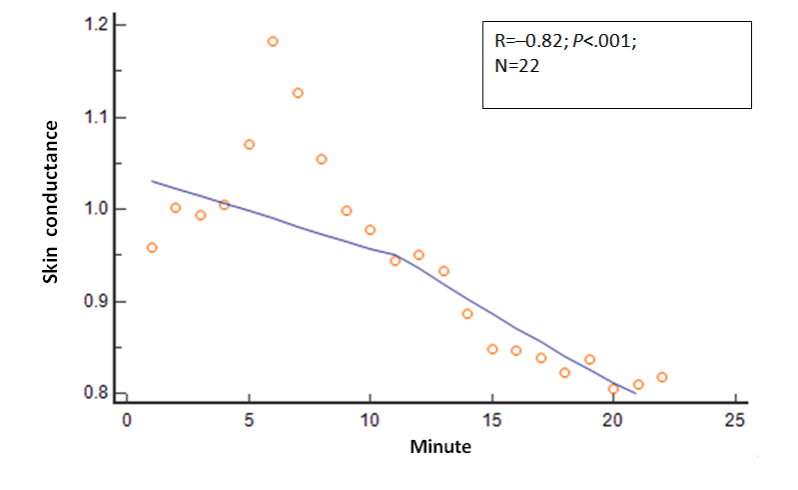
Relationship of the mean skin conductance with time. As the session progressed, the mean skin conductance values steadily decreased.

**Figure 6:**
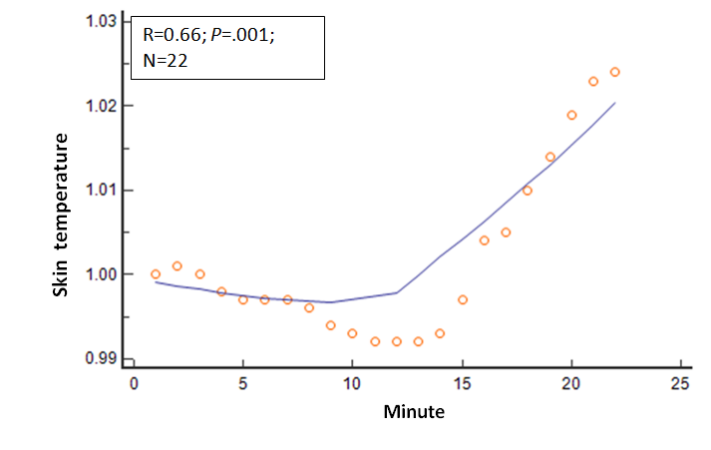
Relationship of the mean skin temperature with time. As the session progressed, the mean skin temperature values steadily increased.

**Figure 7:**
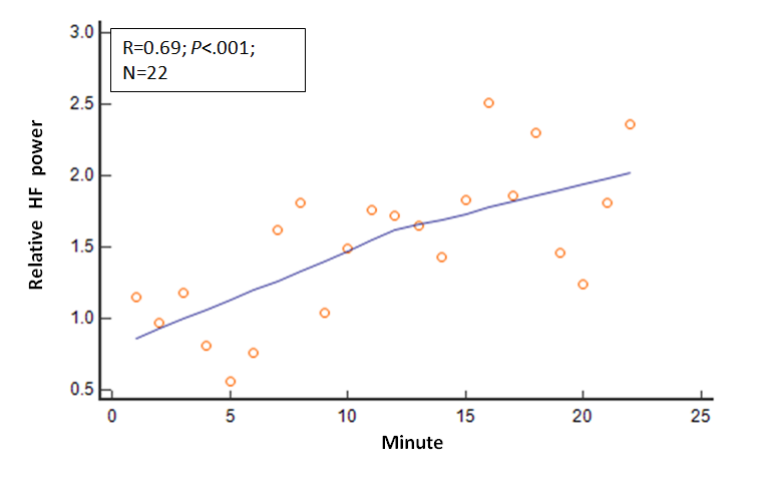
Relationship of the mean relative HF power with time. As the session progressed, the mean relative HF power values steadily increased. HF: high frequency.

**Figure 8:**
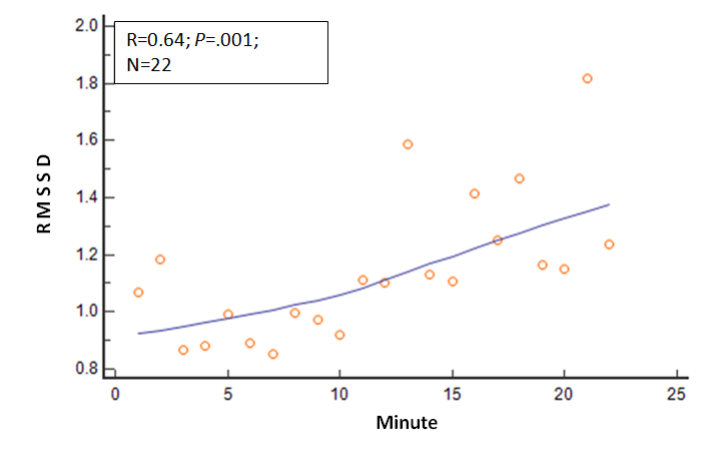
Relationship of the RMSSD with time. As the session progressed, the RMSSD steadily increased. RMSSD: root mean square of successive cardiac interbeat time interval differences.

Several other physiologic variables showed significant changes in the 22 mean values as the session duration in minutes progressed. The R values and *P* values for these physiologic variables relative to session progression time in minutes (correlation analysis: mean variable values=session minutes) were as follows: EMG, R=−0.80 and *P*<.001; expired carbon dioxide, R=0.67 and *P*=.001; respiratory rate, R=−0.68 and *P*<.001; skin conductance, R=−0.82 and *P*<.001; skin temperature, R=.66 and *P*=.001; relative HF power, R=0.69 and *P*<.001; relative VLF power, R=−0.38 and *P*=.08; and RMSSD, R=0.64 and *P*=.001. These relationships are shown in Figures 2-8.

When compared with prehypnosis (min 1-4), BIS, EMG, respiratory rate, skin conductance, and relative VLF power values decreased during induction and postinduction hypnosis, whereas expired carbon dioxide and relative HF fraction values increased during induction and postinduction hypnosis (min 7-22; Table 2). When compared with prehypnosis (min 1-4), BIS, EMG, respiratory rate, skin conductance, and relative VLF power values decreased during postinduction hypnosis, whereas expired carbon dioxide, skin temperature, relative HF power, and RMSSD values increased during postinduction hypnosis (min 16-22; Table 3).

**Table 2:** Significant hypnosis-associated physiologic changes (min 7-22).

Physiological measures	Prehypnosis (min 1-4; n^a^=12), mean (SD)	Hypnosis (min 7-22; n^a^=48), mean (SD)	*P* value	Cohen *d*
Bispectral index	96.5 (1.7)	85.6 (5.7)	<.001	2.6
Electromyography (decibel)	50.0 (3.8)	41.4 (2.4)	<.001	2.7
Relative respiratory rate	0.999 (0.11)	0.781 (0.10)	<.001	2.1
Relative skin conductance	0.990 (0.04)	0.906 (0.19)	.006	0.6
Relative very low frequency power	0.892 (0.60)	0.525 (0.38)	.07	0.7
Relative expired carbon dioxide	0.999 (0.02)	1.073 (0.08)	<.001	1.3
Relative high frequency power	1.026 (0.27)	1.743 (1.17)	<.001	0.8

^a^Data points (number of measurements).

**Table 3:** Significant postinduction hypnosis-associated physiologic changes (min 16-22).

Physiological measures	Prehypnosis (min 1-4; n^a^=12), mean (SD)	Postinduction hypnosis (min 16-22; n^a^=21), mean (SD)	*P* value	Cohen *d*
Bispectral index	96.5 (1.7)	82.9 (4.1)	<.001	4.3
Electromyography (decibel)	50.0 (3.8)	41.2 (1.9)	<.001	2.9
Relative respiratory rate	0.999 (0.11)	0.760 (0.09)	<.001	2.4
Relative skin conductance	0.990 (0.04)	0.825 (0.21)	.002	1.1
Relative very low frequency power	0.892 (0.60)	0.467 (0.35)	.04	0.9
Expired carbon dioxide	0.999 (0.02)	1.068 (0.08)	<.001	1.2
Relative skin temperature	0.999 (0.00)	1.014 (0.03)	.04	0.7
Relative high frequency power	1.026 (0.27)	1.933 (1.28)	.005	1.0
Relative root mean square of successive cardiac interbeat time intervals	0.999 (0.17)	1.357 (0.70)	.04	0.7

^a^Data points (number of measurements).

### Significant BIS Correlations With Other Autonomic Variables

Correlation analyses of the 66 BIS values during minutes 1-22 showed multiple significant associations with other physiological variables. When BIS values decreased, EMG, respiratory rate, skin conductance, and relative LF power values decreased (Table 4). When BIS values decreased, HF power, relative HF power, RMSSD, and SDNN values increased (Table 4). Multivariate linear regression analysis showed that the 66 BIS values during minutes 1-22 were simultaneously and independently associated with EMG (*P*<.001), skin conductance (*P*<.001), and relative LF power (*P*=.03), with a total R^2^=0.71. HF power had positive univariate associations with SDNN (R=0.78; *P*<.001) and RMSSD (R=0.85; *P*<.001).

**Table 4:** Significant bispectral index correlations with other autonomic variables (min 1-22).

Physiological measures	R value	*P* value
Electromyography	0.76	<.001
Respiratory rate	0.35	.004
Skin conductance	0.57	<.001
Relative low frequency power	0.32	.01
High frequency power	−0.41	<.001
Relative high frequency power	−0.53	<.001
Root mean square of successive cardiac interbeat time intervals	−0.32	.009
SD of cardiac interbeat (normal-to-normal) time intervals	−0.26	.04

## Discussion

### Principal Findings

The goal of this formative and feasibility assessment was to determine whether hypnosis could produce sustained reductions in BIS values. The results demonstrate that hypnosis produced sustained reductions in BIS values in healthy volunteers and that the BIS values steadily decreased linearly as the hypnosis session progressed. Another goal of this formative assessment was to organize and evaluate a process that could identify significant changes and correlations between BIS values and other measurements of physiologic relaxation. In implementing the complex and coordinated data collection process described in the *Methods* section, we were able to identify significant changes and associations between BIS values and EMG, respiratory rate, skin conductance and temperature, expired carbon dioxide, and HRV values. These findings indicate that when mental relaxation occurs, physical relaxation occurs simultaneously. These associations also demonstrate that hypnosis fostered a state of mental and physical relaxation in this small group of healthy volunteers and indicates that the process could be feasibly applied to a larger homogeneous group of trauma center physicians and nurses.

### Changes in BIS Values During Hypnosis

As the hypnosis session progressed, the BIS values progressively decreased. Compared with the prehypnosis state, BIS values were lower during hypnosis induction and even lower during postinduction hypnosis. The mean BIS values during hypnosis induction and postinduction were similar to the BIS values reported during stage I sleep [74]. The BIS reductions that we found during hypnosis are corroborated by another similar investigation by Almeida-Marques [8]. In their study, the mean BIS values before hypnosis were also 97, but they averaged 77 in the hypnotic state, which is lower than that in this investigation. However, their sample size was larger and consisted of patients with fibromyalgia, and they employed a hypnotherapist for their sessions rather than using a prerecorded hypnosis script [8]. Supplementary evidence for BIS reductions is the evidence provided during investigations of participants watching relaxing videos [10] or trying relaxing-guided imagery [11]. The brainwave physiology of BIS reductions, and similarity to stage I sleep, implies that such participants were in a relative state of cognitive or mental relaxation [73-75].

### Changes in HRV During Hypnosis

Hypnosis was associated with changes in HRV in this assessment. In particular, relative HF power and RMSSD values increased, whereas relative VLF power values decreased. The influence of hypnosis on HRV has been explored by several investigators [16-21]. With hypnosis, LF power has been shown to decrease [16-18], whereas HF power has been demonstrated to increase [17,19,20]. Accordingly, LF/HF power has been noted to decrease [16,21]. During hypnosis, RMSSD, SDNN, and the interbeat interval mean and SD have been shown to increase [19,20].

Contrary to other investigations [16-21], we did not observe any significant decreases in LF and LF/HF power or increases in SDNN and the interbeat interval in this assessment. This discrepancy could be related to the very small number of participants in this study. HRV changes in a larger sample would be more revealing. Similar to other investigations, the increases seen in relative HF power and RMSSD values suggest that there was an increase in parasympathetic neural activation during hypnosis [13,17,19,20]. The interpretation of the decrease in relative VLF power during hypnosis is somewhat uncertain. Two recent publications specifically stated that the association between VLF power and autonomic neural function is unclear [76,77]. However, both investigations demonstrated that nonrandom eye movement sleep was associated with reductions in VLF power, when compared with the awake state. Furthermore, the reductions in VLF power in these studies were associated with reductions in LF and LF/HF power, suggesting that reductions in VLF likely imply that a reduction in sympathetic neural activity existed.

### Changes in Other Autonomic Variables During Hypnosis

This assessment provides evidence that hypnosis is associated with increases in expired carbon dioxide and skin temperature and decreases in EMG, respiratory rate, and skin conductance values. Nearly all studies investigating the effects of hypnosis on skin temperature involved focused attempts to warm or cool. Only one other investigation demonstrated that neutral hypnotic induction was associated with passive significant increases in skin temperature in pediatric patients [36]. In this assessment, we were able to demonstrate passive significant increases in skin temperature with neutral hypnotic induction in healthy adult volunteers. As in this assessment, 2 other investigations, one in patients with fibromyalgia and the other in healthy adults, demonstrated a decrease in skin conductance during hypnosis [8,29]. The increases in skin temperature and reductions in skin conductance are consistent with a decrease in sympathetic neural activity during hypnosis.

The finding that hypnosis was associated with an increase in expired carbon dioxide is relatively novel. Only 1 study, published more than 30 years ago, has investigated expired carbon dioxide measurements during hypnosis [30]. In that study, the authors compared the results of 27 patients with hyperventilation syndrome with those of 10 healthy controls, who were hospital workers [30]. The results demonstrated that both the patient and control groups had increased expired carbon dioxide and decreased respiratory rates during the deep relaxation phase of hypnosis, similar to the findings of this assessment. Other investigations of healthy adult volunteers also demonstrated that the respiratory rate significantly decreased during hypnosis, similar to the findings of this assessment [31,32]. Although 1 study was performed in France and the other in the United States, the authors reported identical decreases in respiratory rates from 18 to 14 breaths per minute [31,32]. Other hypnosis investigations demonstrated significant reductions in facial EMG measurements in healthy volunteers during hypnosis, as we did in this assessment [33-35]. The increases in expired carbon dioxide values and decreases in the respiratory rate and EMG values are consistent with the onset of relaxation during hypnosis.

### Significant Correlations of BIS Values With Other Autonomic Variables

A unique aspect of this assessment is exploring the associations of BIS values with other autonomic measurements. All other investigations that measured BIS during hypnosis reported other measures of physiologic relaxation as coparameters, not as correlations, or were confounded. We identified several significant associations between BIS values and other physiological variables over the 22 session minutes. When BIS values decreased, EMG, respiratory rate, skin conductance, and relative LF power values decreased. However, as BIS values decreased, HF power, relative HF power, RMSSD, and SDNN values increased. The simultaneous reductions in BIS, respiratory rate, and EMG values suggest that lower BIS values connote the presence of a state of physiologic relaxation. The concomitant decreases in BIS values and increases in HF power, relative HF power, RMSSD, and SDNN values imply that BIS reductions are associated with increased parasympathetic neural activation [13]. The large positive associations that we found between HF power with SDNN and RMSSD further support the notion that increased parasympathetic neural activity parallels reductions in BIS values. The concurrent reductions in BIS, skin conductance, and relative LF power values suggest that sympathetic neural activation is decreased [13]. Multivariate linear regression analysis showed that BIS values during the 22-minute session were simultaneously and independently associated with EMG, skin conductance, and relative LF power. These findings are consistent with the notion that reductions in BIS values are principally related to a combined state of relaxation and blunted sympathetic neural responses; however, multiple analyses also suggest that parasympathetic neural activation is also present.

### Duration of the Experimental Procedure

More than 20 publications were cited at the beginning of this manuscript regarding the impact of hypnosis on relevant physiological processes. Of those manuscripts, 8 provided the duration (in minutes) of the control period plus the hypnosis phase. The total duration was 10-30 minutes in 4 manuscripts [1,3,19,36] and 40-45 minutes in the other 4 manuscripts [17,29,30,33]. Hypnosis audiotapes were used in 4 of the investigations. Of relevance, 2 meta-analyses of randomized controlled trials provided substantial documentation regarding the duration of hypnosis sessions in the included studies [78,79]. The duration of the hypnosis period was documented to be 3-20 minutes in 14 studies and 23-30 minutes in 8 trials. Hypnosis audiotapes were used in 8 of these 22 studies. The aforementioned evidence lends credibility to the duration of this experimental design and the use of hypnosis audiotapes.

### Limitations

The principal limitation of this formative assessment is the small number of participants; however, the statistical significance of the findings is compelling. Another limitation is that the authors did not compare results between the participants because 3 participants are an insufficient number to compare. Comparisons between age, sex, and other epidemiological information is considered thought provoking in a larger sample. A posthypnosis observation period was not evaluated to determine if the hypnosis-associated physiologic changes were sustained for any period afterward, which would be informative. Finally, the authors did not obtain data on medications, supplements, or caffeine intake, which could affect participants’ physiological variables in a resting state.

### Conclusions

The preliminary formative assessment results indicated that the 2 assessment objectives were met. These findings suggest that hypnosis may manifest as a state of mental and physical relaxation, enhance parasympathetic nervous system activation, and attenuate sympathetic nervous system activation, observations that were associated with reductions in BIS values. They also imply that reduced BIS values were associated with a state of physiologic relaxation, increased parasympathetic nervous system activity, and decreased sympathetic nervous system stimulation. In addition to the findings of improvements in participant well-being in our BIS neurofeedback study [73], this evaluation lends further support to the notion that the BIS monitor may be a valid device for use in neurofeedback investigations. Finally, the BIS monitor may subsequently be demonstrated to be considered as a *hypnometer* for continuously measuring hypnotic depth, as proposed by the former Harvard University investigator Solomon Gilbert Diamond [19]. The findings of this preliminary formative evaluation indicate that this hypnosis model may be useful for assessing autonomic physiological associations with changes in BIS values. These observations from this exploratory process motivated us to proceed with a larger and similarly designed investigation that will include trauma center nurses and physician participants.

## Abbreviations

BIS: bispectral index
EEG: electroencephalography
EMG: electromyography
HF: high frequency
HRV: heart rate variability
LF: low frequency
RMSSD: root mean square of successive cardiac interbeat time intervals
SDNN: SD of cardiac interbeat (normal-to-normal)
VLF: very low frequency
